# Thin Layer Buckling in Perovskite CsPbBr_3_ Nanobelts

**DOI:** 10.1021/acs.nanolett.1c00962

**Published:** 2021-06-28

**Authors:** Emma H. Massasa, Rotem Strassberg, Amit Vurgaft, Yaron Kauffmann, Noy Cohen, Yehonadav Bekenstein

**Affiliations:** † Department of Materials Science and Engineering, Technion − Israel Institute of Technology, Haifa 32000, Israel; ‡ The Solid-State Institute, Technion − Israel Institute of Technology, 32000 Haifa, Israel; § The Nancy and Stephen Grand Technion Energy Program, Technion − Israel Institute of Technology, Haifa 32000, Israel

**Keywords:** thin layer buckling, lead halide perovskites, mechanical deformation, bend contrast, nanobelts, energy materials

## Abstract

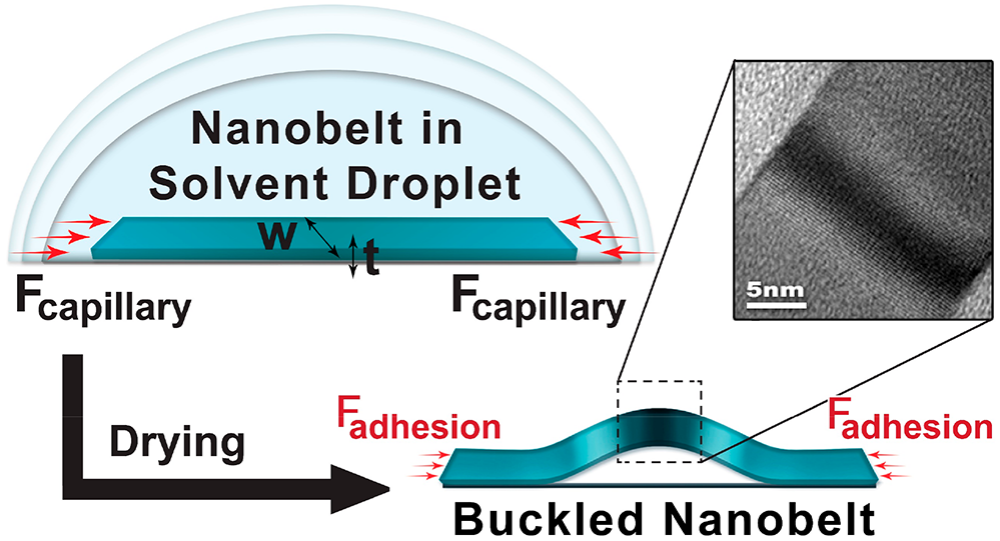

Flexible semiconductor materials, where structural fluctuations and transformation are tolerable and have low impact on electronic properties, focus interest for future applications. Two-dimensional thin layer lead halide perovskites are hailed for their unconventional optoelectronic features. We report structural deformations via thin layer buckling in colloidal CsPbBr_3_ nanobelts adsorbed on carbon substrates. The microstructure of buckled nanobelts is determined using transmission electron microscopy and atomic force microscopy. We measured significant decrease in emission from the buckled nanobelt using cathodoluminescence, marking the influence of such mechanical deformations on electronic properties. By employing plate buckling theory, we approximate adhesion forces between the buckled nanobelt and the substrate to be *F*_adhesion_ ∼ 0.12 μN, marking a limit to sustain such deformation. This work highlights detrimental effects of mechanical buckling on electronic properties in halide perovskite nanostructures and points toward the capillary action that should be minimized in fabrication of future devices and heterostructures based on nanoperovskites.

Low dimensional metal lead halide perovskites pose excellent optoelectronic properties such as high quantum yield, narrow and tunable emission, and affordable chemical synthesis that do not require costly inorganic shelling.^1−8^ Contrary to typical semiconductors, the halide perovskites lattice is considered soft^9−22^ with long chemical bond lengths. Affecting vibrational properties,^9,14,15^ electron phonon interactions,^14,23^ supporting exciton polaritons,^14^ as well as facile ion diffusivity^10,12,13,17,18,20−22^ and structural transformation.^10^ Understanding structure deformation in low dimensional perovskites and its effects on the optoelectronic properties is of fundamental and technological importance. Studies relating structural transformations and pressure of bulk crystal and thin films have been considered in recent years.^24−33^ Some structural deformation can modify the electronic band structure and thereby modulate the optoelectronic properties.^25−28,30−32,34−38^ For example, Zhang et al.^31^ simulated the change of the band structure of methyl-ammonium lead iodide perovskite as a result of external strains, and Wang et al.^30^ reported the effect of pressure on the structure of MAPbBr_3_ and its influence on the optic and electronic properties. In a review by Jaff et al.,^39^ the effects of compression on the structure of the perovskites and hence the optical and electronic properties of the material were presented and changes from absorption and photoluminescence (PL) emission to metallization were discussed. In the case of low dimensional anisotropic nanocrystals, the situation is more complex due to the strong asymmetry in the crystal’s dimensions.^40,41^ Recent developments in colloidal synthesis of lead halide nanocrystals include growth of nanocubes,^42^ nanosheets,^43^ nanowires,^44^ nanoplates,^45^ and nanobelts.^46^ In this study, we focus on colloidally synthesized CsPbBr_3_ with nanobelt shape, in which the thickness is of a few unit cells and the length is of hundreds of nanometers up to a few micrometers. A colloidal suspension of the nanobelts is deposited onto carbon covered substrates and let dry. Through transmission electron microscopy (TEM) characterization, structural deformation is observed in the form of electron contrast bands across the perovskite nanobelts. Analysis of experimental high-resolution TEM (HRTEM) micrographs accompanied by multislice computer simulations were used to confirm that the contrast bands are structural deformations in the form of thin layer buckling and forces leading to these phenomena are extracted.

## Contrast Characterization

Colloidal CsPbBr_3_ perovskite nanobelts were synthesized and cleaned as detailed in the Supporting Information (SI). In short, CsBr and PbBr_2_ were dissolved in acetone with oleic acid and oleyl amine as ligands for a certain amount of time, then centrifuged and redispersed in hexane. This synthesis was based on a nanowire synthesis done by Liu et al.^47^ with changes in the salt ratio, centrifuge speed, and redispersion solvent. Optical absorption and photoluminescence spectra of the nanobelts were recorded at room temperature and are shown in Figures S1a,b, respectively. The nanobelts absorption spectrum includes the first and second exciton transitions at 519 and 367 nm, respectively, while the PL spectrum shows an emission peak at 522 nm in agreement with weakly quantum confined excitons.^48−50^

**Figure 1:**
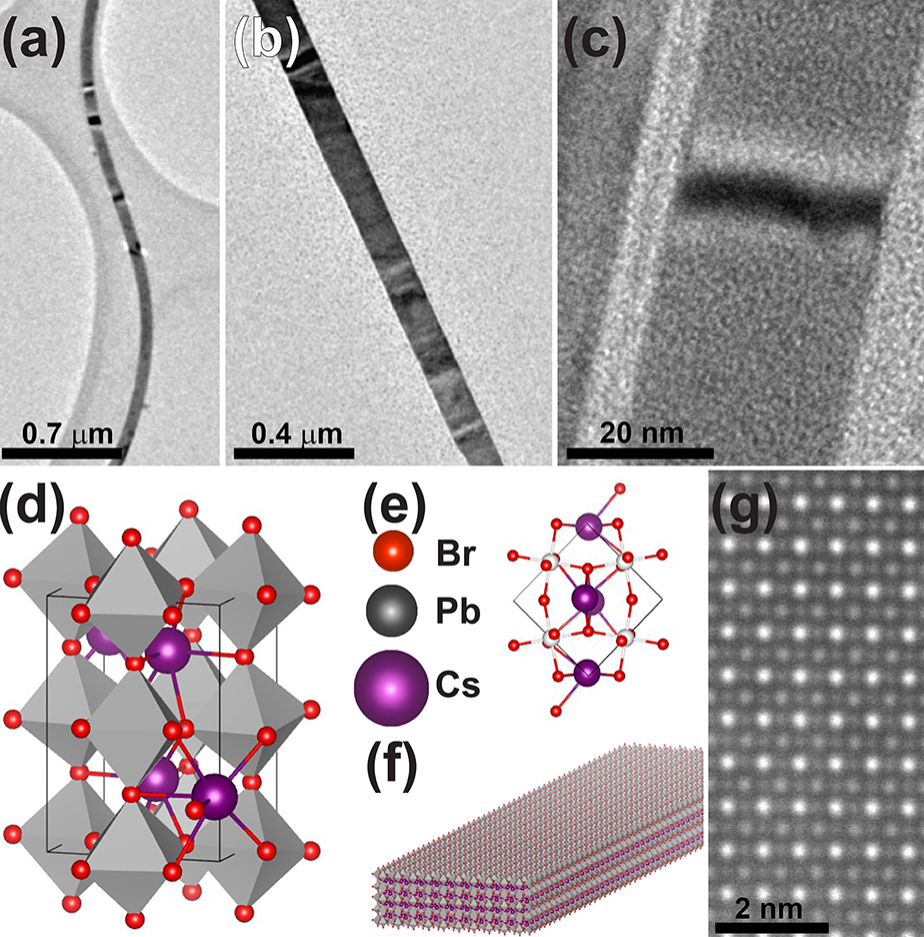
Electron microscopy characterization of the CsPbBr_3_ nanobelts. (a–c) Low-magnification TEM micrographs of CsPbBr_3_ nanobelts with 10–200 nm width and up to a few micrometers in length. High/low contrast strips across the nanobelts are investigated as potential structural deformation of the perovskite nanobelts. (d) Model of CsPbBr_3_ extended unit cell with tilted octahedral typical to the orthorhombic structure. (e) CsPbBr_3_ unit cell from a [001] view. (f) Schematics of a nanobelt profile not to scale, typical thickness of ∼5 nm where width and length are tens and hundreds of nanometers, respectively. (g) Aberration corrected HRSTEM micrograph of the perovskite nanobelts showing typical column along the [001] zone axis similar to that presented in (e).

Indeed, TEM and atomic force microscopy (AFM) characterizations of the nanobelts (Figure 1a–c, and Figure S4) depict lateral dimensions of a few microns in length, 10–200 nm in width, and with a thickness of 2.5–10 nm on the scale of the Bohr exciton radius,^51^ (Figure 1f). CsPbBr_3_ nanobelts present orthorhombic crystal structure (Figure 1d) similar to nanowires^44^ as determined by selected area diffraction (SAD) (shown in Figure S2), X-ray diffraction (XRD) (Figure S3), and aberration corrected HRSTEM as shown in Figure 1g, which is schematically shown in Figure 1e.

TEM micrographs of CsPbBr_3_ nanostructures shown in Figure 1a–c depict a dark-bright strip feature across the nanobelt. Electron micrograph contrast bands in thin crystal lattices are associated with (1) variations of crystal thickness and (2) thin layer buckling (abrupt change in height due to crystal ripples).^52^ Here, we attribute the contrast bands to perovskite nanobelts buckling and not variation in the nanobelts thickness. This deduction is supported by four different electron microscopy methods: (1) selected orientation dark-field (DF) imaging, (2) specimen tilting, (3) a dynamic measurement, and (4) Quantitative TEM simulations, all detailed in this study.

**Figure 2:**
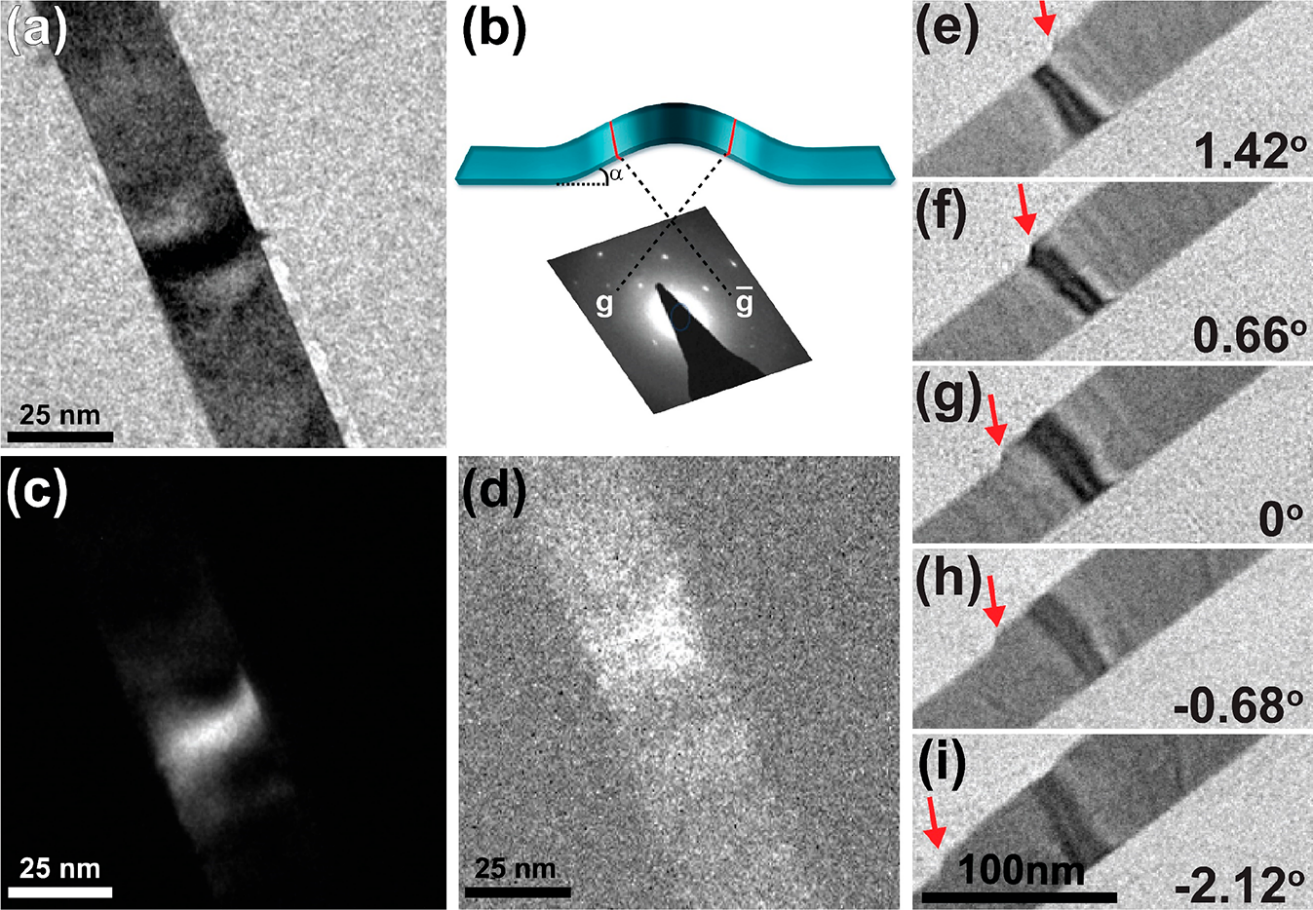
TEM characterization of buckled nanobelts using selected orientation in dark-field mode and tilting experiment. (a) TEM micrograph of CsPbBr_3_ nanobelt indicating contrast strips across the nanobelt. (b) A sketch of the characterization technique. Buckled nanobelt (with an α-buckle angle) corresponding diffractogram depicting opposite reflections. Electrons scattered from these points form complementary dark-field micrographs as seen in (c,d), depicting variation in contrast and position of contrast band due to the buckled perovskite structure. (e–i) Movement of the bend contrast with the change of the sample tilt. (e) 1.42°, (f) 0.66°, (g) 0°, (h) −0.68°, and (i) −2.12°; the red arrows indicate a fixed location on the nanobelt, showing a pure amplitude contour.

In order to study the microstructure of the buckled nanobelts, we used a modified text book characterization technique,^52^ which we name selected orientation dark-field imaging (SODFI). In this method, one selects two opposite reflections of a selected area diffraction pattern. Each reflection corresponds to a specific angle of transmitted electrons scattered from the crystal. By selecting opposite reflections, we ensure the angles are equal but opposite in sign. Next, an aperture is moved to capture a dark-field micrograph from each of these orientations. Figure 2a shows a micrograph of a buckled nanobelt. The sketch in Figure 2b demonstrates the principles of the SODFI characterization technique. In the case of a buckled nanobelt, when a reflection is chosen for a DF imaging only electrons scattered from a specific angle are collected by the detector. So, when imaged two opposing sides of the buckled nanobelt present themselves as high contrast regions. This is demonstrated in Figure 2c,d showing higher contrast on opposite sides of the buckled area.

In order to reassure the bands are indeed buckled areas of the nanobelts and not local variations in the thickness of the crystals, we conducted an experiment where we tilt the sample in respect to the electron beam while monitoring the change in contrast of the bands. This is a text book procedure to differentiate between bend and thickness contrast; if a change in relative tilt will cause movement of the contrast, then the contrast is a pure amplitude contrast and not a result of mass-thickness contrast.^52^ In Figure 2e–i, we detect clear movement of the contrast of the buckled area, as we change the tilt of the sample. In addition, we see a correlation between the direction of the tilt and the direction in which the contrast moves. Another indication of a bend contrast is if one detects movement of the contrast (without tilting the sample).^53−62^ The movement of the contrast was examined during a minute long electron imaging exposure. Figure S6 depicts a series of micrographs of the same bend contrast taken approximately a minute apart. The distance between two contrast bends on the same nanobelt was measured to be 51 and 39 nm. While thickness variations typically display static electron micrograph contrast bands,^63^ a crystal ripple (buckled structure) could be more dynamic.^53−62^ This dynamic, typically referred to as “jittering effect”, is a consequence of local charging accumulation caused by the electron beam.^55,56,58,61,62^ A schematic illustration of proposed charging in buckled nanobelts is demonstrated in Figure S6b,c. When the electron beam (*I*_0_) penetrates the sample, the specimen atoms interact with it, creating elastic (*I*_e_) and inelastic (*I*_i_) collisions. In this process, Auger and secondary electrons (*I*_s_) might be emitted from the specimen causing an accumulation of positive charge on its surface.^64−68^ In insulating and semiconducting materials, the conductive amorphous carbon film on the TEM grid prevents charging effects.^64,66,68^ However, in the case of a crystal ripple, or a buckled area which does not have direct contact to the conductive substrate, accumulation of charges is possible. Charging effects will modify the formed image and their discharge will cause movements, blurring, and focus modifications, which may be displayed in a jittering effect as seen in the buckled nanobelts.^64,65,68^ Such jittering was noticed both in TEM and scanning electron microscopy (SEM) experiments (Figure S5).

**Figure 3:**
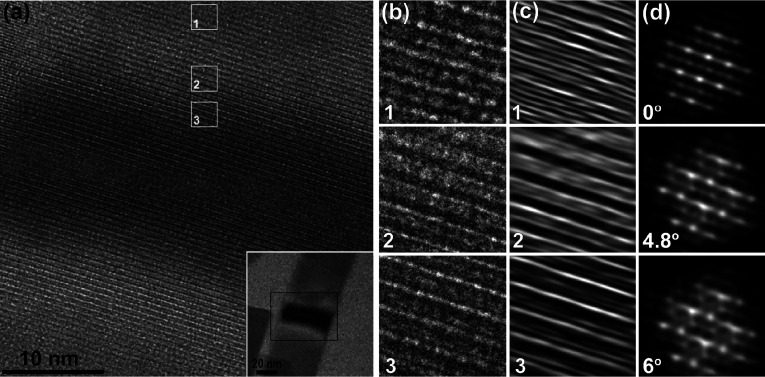
HRTEM study of the buckled nanobelt. (a) HRTEM of the contrast strip showing details of the microstructure with higher contrast at the edges and lower contrast at the middle, at the right bottom corner is an inset of low-magnification micrograph of the buckled area. (b) Areas 1–3 blown up of the lattice fringes, matching selected areas in the contrast strip, (c) FFT filtered images of (b) 1–3 demonstrating that the contrast difference is due to change in lattice fringes density. (d) Quantitative TEM simulated images, based on a multislice algorithm of tilted CsPbBr_3_ crystals (α = 0, 4.8, 6°). The simulation demonstrates that the tilting reduces contrast of some lattice lines.

In order to better understand how lattice orientation of buckled thin layer halide perovskite results in a contrasted band in the nanobelt TEM micrograph, we analyzed numerically modeled high-resolution TEM data. Figure 3a (inset) shows a high-magnification (low-magnification) typical microstructure of the buckled nanobelt depicting symmetric white-black-white contrast pattern. We note in passing that other typical “inverted color patterns” may also be observed (as seen in Figure 1c and Figure S5). Figure 3b exhibits blowups of selected contrast band areas (marked (1–3) in Figure 3a) where variations in lattice fringe thickness and density along the ripple are observed. Specifically, along the contrast band areas marked by (1) show thin fringes (0.1 nm) with small distance separation (0.16 nm). Thicker fringes (0.3 nm) are denoted by (2) and last (3) areas appear with thin fringes (0.16 nm) but larger distance separation (0.58 nm). The overall change in fringe density is clearly seen in the FFT filtered images (Figure 3c (1–3)). We now use our two observations as follows: (1) Band contrast is associated with tilted perovskite lattice, and (2) HRTEM micrographs depict variations in lattice fringes density across the bands. To simulate electron transmission through the perturbed perovskites lattices, we use Quantitative STEM/TEM (QSTEM) simulation package for electron transmission calculations based on a multislice algorithm^69^ (see SI for details). Figure 3d (1–3) depicts intensity simulations of scattered electrons when transmitted through a CsPbBr_3_ crystal. When the angle between the sample and the electron beam (90-α) is varied (α = 0–6°), a general blurring of the pattern occurs. This is evident by the widening of lattice fringes and the reducing of the overall contrast of the image; we will note that the simulation presented in Figure 3d does not consider changes in the focus as a result of the height change in the buckled area.

**Figure 4:**
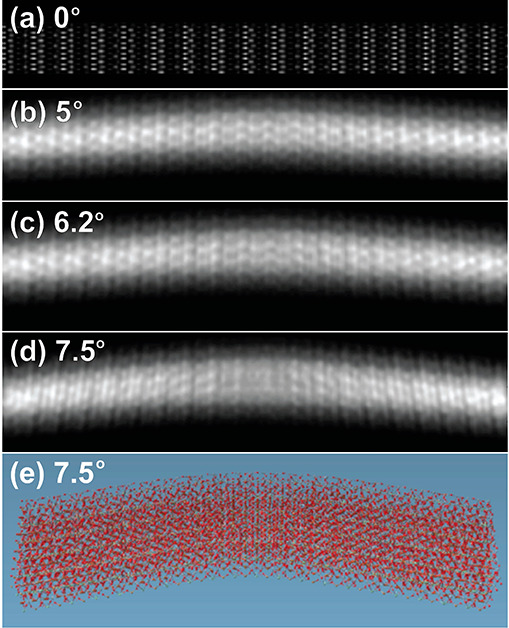
Simulated TEM images using multislice algorithm of a series of bent nanobelts in small angles. The simulation is based on scattering from a single crystal atomistic model of a perovskite nanobelt. It shows a development of a contrast strip feature in a buckling angle of more than 7°. (d) A contrast difference in the center (blurry) and edge (change in lattice fringes) of the simulated bent nanobelt, matches the observed bend contrast. The nanobelt models are structured using atomistic perovskite unit cell expanded to a cross section of the nanobelt. The simulations consist of series of angles (a) 0°, (b) 5°, (c) 6.2°, and (d) 7.5°.

Therefore, to model the extent of buckling in perovskite nanobelts simulations based on an atomistic model of bent perovskite nanobelts via a commercially available numerical simulation software (Samson, see SI for details) were carried out. It should be noted that the simulated buckled nanobelt obtained in Figure 4 is independent of the TEM micrographs received in our experiments. A series of crystals models of a buckled nanobelt with different buckling angles (α) (via Samson software) was used for electron scattering simulation (via QSTEM software).^69^Figure 4a–d and e show the simulated electron micrographs and one example of an atomistic model of buckled perovskite nanobelt used for the simulations, respectively. The simulated micrographs (Figure 4a–d) display a distortion of the lattice image with the increase of α. Figure 4a shows simulation of an unbuckled nanobelt, which is clearer than the simulation of a buckled nanobelt due to (1) change in the angle between the electron beam and crystal surface, and (2) change in the height of the buckled area. We note that both of these will influence the focus and contrast. In Figure 4d, an onset in the lattice fringe periodicity is detected with the blurred lattice fringes at the middle of the ripple contrast. The effect of the ripple is pronounced from an angle of 7.5° which shows a resemblance to the contrast patterns seen in measured TEM and HRTEM micrographs (Figures 1–3). The atomistic modeling simulation of a buckled crystal for simulating its electron micrograph achieves two goals: (1) Confirms that the contrast phenomenon is indeed a crystal ripple in perovskite nanobelts. (2) Gives a lower limit for a buckling angle of 7.5° to allow observation of contrast features from buckled perovskite nanobelts under our experimental conditions.

## Buckling-Mechanical Model

Probable forces that may lead to buckling of nanobelts are generated by the drying of colloidal suspensions and resulting capillary forces. At the nanoscale, capillary forces play an important role^70−75^ due to high surface-to-volume ratios and the minimization of surface energies upon creation of interfaces. A colloidal suspension of perovskite nanobelts is drop casted on a TEM carbon grid and let dry in open air. Capillary forces of the drying solvent strain nanobelts that are adsorbed on the grid. During the drying process, the capillary action leads to the generation of a compressive force that is applied on the nanobelt. Once a critical force is reached, the nanobelt buckles. Once the solvent completely dries, the buckled configuration of the nanobelts is maintained by adhesion forces between the nanobelt and the substrate (Figure S7). The adhesion has a normal and tangential components, which adhere the nanobelt to the surface and maintain the buckling, respectively. By employing buckling theory,^76^ we can estimate this adhesion force from plate buckling theory (*F*_critical_ ≅ *F*_adhesion_).
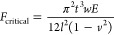
1where υ = 0.3^77^ and *E* = 28 GPa^77^ are the Poisson’s ratio and the Young’s modulus of the nanobelt, respectively, and *t*, *w*, and *l* are the thickness, width, and length of the nanobelt at the buckled segment, respectively. The width and the length are measured from TEM micrographs, while the thickness is estimated to be ∼9 nm (see SI for justification).

We calculate *F*_adhesion_ = 0.12 ± 0.06 μN where the error stems from the uncertainty in the thicknesses of the nanobelts and from the orthorhombic crystallographic structure which is not taken into account in our model. From the above analysis, we find that CsPbBr_3_ nanobelts adhere to amorphous carbon surfaces with force at the scale of 0.12 μN, which is in the typical adhesion force scale.^78−84^ Xie et al. discussed the influence of the van der Waals forces on the stable buckled state and showed that the balance of the energies leads to a specific height of buckled structures.^38^ One has to consider also that organic ligands that cover the nanobelt’s surfaces may contribute to these attracting forces. Any future device or application that will require processing of such thin colloidal perovskites cannot ignore these adhesion and capillary forces in order to avoid buckling phenomena.

## Buckling Analysis

**Figure 5:**
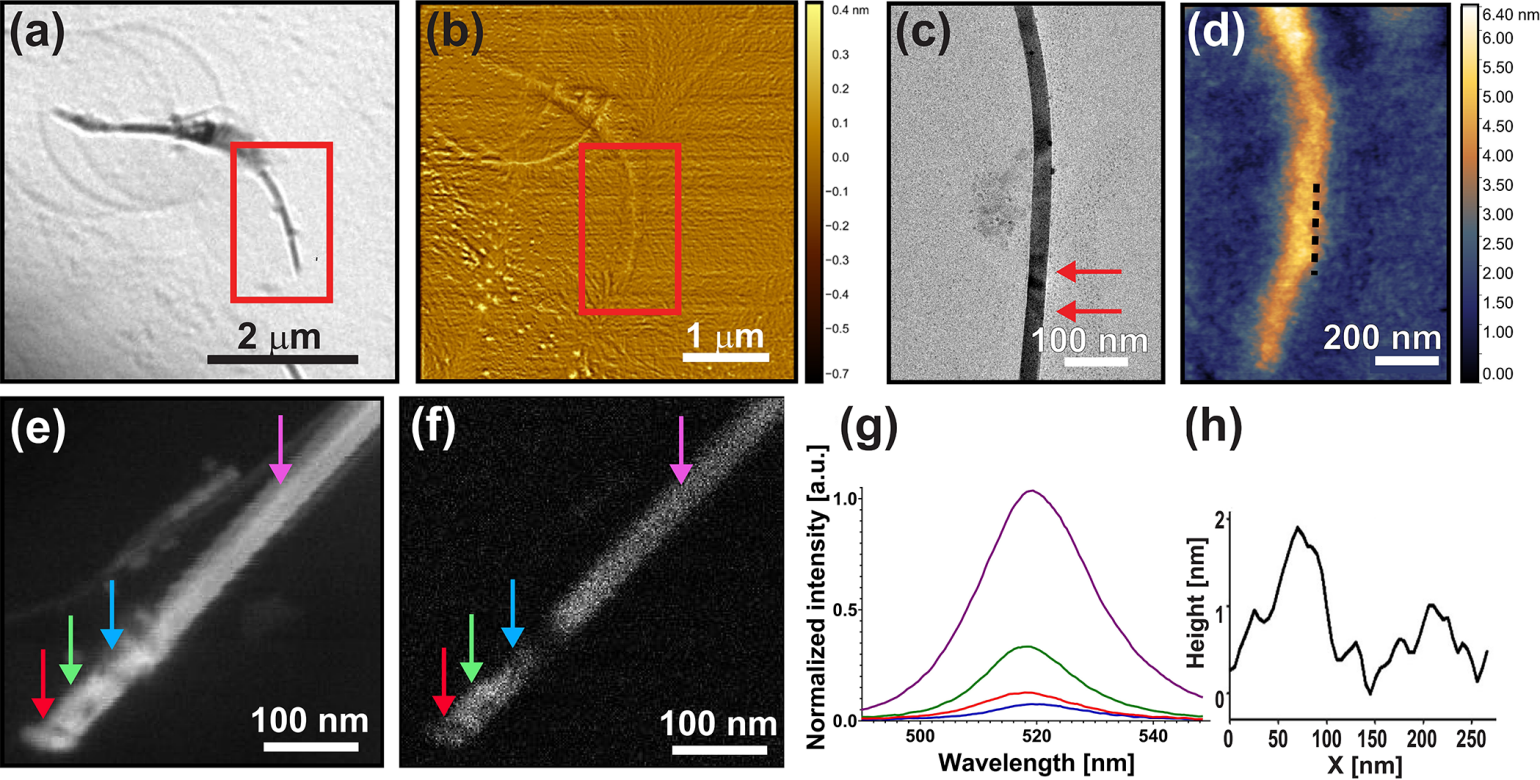
Buckling topography correlations and characterization. (a) TEM micrograph of a buckled nanobelt. (b) AFM micrograph of the same nanobelt as shown in (a). (c) TEM micrograph of the buckled area, marked in (a), indicated with the red arrows. (d) AFM micrograph of the marked area in (b) showing the same buckled nanobelt as seen in (c). (e) SEM micrograph of a buckled nanobelt, (f) CL micrograph of the same nanobelt in (e), the arrows in (e,f) indicate the locations of the CL measurements. (g) PL spectra of the marked locations in (e,f). (h) Buckling topography measurement of the dashed black line marked in (d).

To characterize the morphology of buckled nanobelts, a correlation between TEM micrograph and an AFM scan was implemented using a finder grid for locating specific buckled nanobelt in the TEM and then relocating it with in an AFM scan; such a measurement is presented in Figure 5 and Figure S8. Figure 5a,b shows TEM and AFM of the same buckled nanobelt. The location of the bend contrast is zoomed in Figure 5c,d. AFM cross section of this area demonstrates topographic variations of 1–3.5 nm (see additional scans in Figure S8). Similar ripple amplitude measurements in the AFM were done in (2T)_2_PbI_4_ halide perovskite by Shi et al.,^34^ showing ripples with a height of 40–50 nm, and in WS_2_–WSe_2_ heterostructures by Xie et al.^38^ showing a ripple height of 1–2 nm with similar scale to our measurements. We note the differences between Shi et al.^34^ and this work are probably due to the nature of the stress applied, which is the heterostructure interface in the work of Shi et al. versus capillary action in this work.

To measure the change in physical properties of the buckled area in CsPbBr_3_ nanobelt SEM cathodoluminescence (CL) was used.^85^ CL enables one to excite specific areas on the nanobelt with nanometer resolution and compare spectroscopic data of buckled and unbuckled areas of the same nanobelt. Figure 5e shows such an experiment where the CL was measured at four locations along the nanobelt, as marked with the arrows. The red and blue arrows point to buckled areas, the green arrow is located between the two buckles, and the purple arrow is located further along the nanobelt, far away from the buckled areas. Figure 5f shows a CL micrograph of the nanobelt with clear intensity differences between the buckled areas (marked with the red and blue arrows) and the rest of the unbuckled nanobelt. The PL emission graphs in Figure 5g corresponds to the positions marked with the arrows which show a significant decrease in intensity between the purple marked location and the red, green, and blue marked locations. We carefully approximate 65–92% decrease in CL intensity which is correlated with the buckled areas. This observation which shows that mechanical buckling that is caused through capillary action dramatically influences the emissive properties of lead halide perovskite nanobelts. The exact mechanism leading modification of electronic properties of buckled perovskites will be investigated in future experiments and may include trap states due to induced defects in the buckled regions, or local strain of the perovskite crystal structure which modifies the excitonic properties. Our findings emphasize the importance of careful processing of colloidal perovskites with intent to minimize readily occurring buckling effects which reduce fluorescence quality.

In conclusion, structural deformations in thin CsPbBr_3_ nanobelts are reported. A band contrast pattern observed in electron microscopy micrographs was analyzed and determined to be thin layer buckling of the perovskite nanobelts. Dark-field micrographs of opposing diffraction reflections indicated different contrast band areas and represented tilted perovskite lattices. This statement is additionally supported by a tilting experiment showing the contrast is a pure amplitude contrast. Correlation of TEM and AFM micrographs of the same buckled area demonstrate topographic variations of 1–3.5 nm. Lead halide perovskite buckled areas showed reduced cathodoluminescence compared to unbuckled areas on the same nanobelt. Since buckling in thin layer CsPbBr_3_ nanobelts detrimentally influence their physical properties, measures should be taken when processing them. A standard plate buckling model was used to estimate capillary action and adhesion forces between the nanobelts and the substrate. We report a lower limit of adhesion to sustain the buckling which may serve future processes involving CsPbBr_3_ nanoperovskite where buckling effects are to be minimized.

## Supporting Information Available

Materials, synthesis, and methods of characterization and simulations. Additional details on the simulation method, the buckling model, and calculations. Figures of absorption, photoluminescence, transmission electron microscopy (TEM) and selected area diffraction (SAD), X-ray diffractometer (XRD) analysis, atomic force microscopy (AFM), and scanning electron microscopy (SEM) (PDF)
