# Supplementary material for: Thin Layer Buckling in Perovskite CsPbBr3 Nanobelts
Source: Nano Lett. 2021 Jun 28;21(13):5564–71. doi: 10.1021/acs.nanolett.1c00962 (PMC8397391; doi:10.1021/acs.nanolett.1c00962)
Supplement: Supplementary file 1 — nl1c00962_si_001.pdf [file nl1c00962_si_001.pdf]

Supporting Information

Thin layer buckling in perovskite $CsPbBr_3$ nanobelts.

Emma H. Massasa[1], Rotem Strassberg[1,2], Amit Vurgaft[2], Yaron Kauffmann[1], Noy Cohen[1] and Yehonadav Bekenstein[1,2,3]*

[1] Department of Materials Science and Engineering, Technion-Israel Institute of Technology, Haifa 32000, Israel [2] The Solid-State Institute, Technion – Israel Institute of Technology, 32000 Haifa, Israel [3] The Nancy and Stephen Grand Technion Energy Program.

Author Address: bekenstein@technion.ac.il

Keywords: thin layer buckling, lead halide perovskites, mechanical deformation, bend contrast, nanobelts, energy materials.

Colloidal $CsPbBr_3$ nanobelt synthesis preparation

Materials: CsBr (99.9%, Aldrich), oleic acid (OA, 90%,Aldrich), $PbBr_2$ (99.999%, Aldrich), oleyl amine (OLA, Aldrich, 70%), hexane (AR, bio labs), acetone (AR, bio labs). All chemicals were used as received.

Methods. Nanobelt synthesis: in a one step process 0.130 gr of $PbBr_2$ and 0.130 gr CsBr were put in a glass vial, with an addition of 10 ml acetone, 1 ml oleic acid (OA) and 0.5 ml oleyl amine (OLA)[1]. The solution was mixed at room temperature and after 1 h and 50 min the nanobelts were precipitated by centrifugation at 8500 rpm for 5 min. Finally, the nanobelts were redispersed in 9 ml of hexane.

Colloidal $CsPbBr_3$ nanobelt Synthesis characterization

UV-Vis photo spectrometer: spectroscopic analysis of the colloidal $CsPbBr_3$ nanobelts was carried out using BioTek Hybrid Multi-Mode Microplate Reader Synergy H1. For the Photoluminescence (PL) measurements the sample was illuminated at 400 nm.

Transmission electron microscopy (TEM): Samples for TEM imaging were prepared by drop casting the nanobelt colloidal solution on a Cu grid with a carbon film at room temperature. In this research we used Transmission Electron Microscope FEI Tecnai G2 T20 S-Twin TEM and High-Resolution Transmission Electron Microscope FEI Titan 80-300 kV FEG-S/TEM. All micrographs in this study were produce with 200 kV acceleration voltage. In the tilting experiment a single tilt sample holder was used, with it the α angle was moved during the nanobelts imaging.

Scanning electron microscopy (SEM): Samples for SEM imaging were prepared by drop casting the nanobelt colloidal solution on an Ag grid with a silica film at room temperature. In this research we used Scanning electron microscopy Quanta 200 FEI E-SEM.

Scanning electron microscopy cathodoluminescence (SEM-CL): CL was collected with a high-resolution SEM (Zeiss Sigma 500) equipped with a field emission gun. A voltage of 20 kV and a SE2 detector were used to produce the SEM micrographs. The CL spectra was collected using a Gatan MonoCL Elite system equipped with a retractable diamond-turned mirror. The measurements were performed with an aperture of 60 μm at analytical gun mode.

Atomic force microscopy (AFM): samples for AFM were prepared by spin coating (1000 rpm) the nanobelt colloidal solution on a Si substrate. In this research we used Asylum MFP-3D AFM in tapping mode at tip resonance frequency of 70 kHz.

Buckling topography analysis- the nanobelt colloidal solution was drop casted on a finder grid, and the nanobelts location was recorded. After the TEM measurement the finder grid was taken to an AFM scan. The grid was fixed to a Si substrate with Cu tape. Specific nanobelts were located and corresponding TEM/AFM micrographs were compared in order to measure the buckling topography.

X-ray diffractometer (XRD): samples for XRD were prepared by precipitating the nanobelts from the colloidal solution by centrifuge and implementing them on a glass substrate. The nanobelts phase characterization was performed on Rigaku Smartlab X-ray diffractometer with Cu Ka radiation, $k\alpha_1 = 1.541$ Å.

Electron beam damage

During electron beam characterization typical damage to $CsPbBr_3$ nanocrystals may include material degradation and decomposition to Pb, CsBr and $PbBr_2$[2–4]. In order to minimize such beam damage, we tried to avoid magnifications higher than 71000x and long exposure times (30 seconds). We can confidently characterize buckling in $CsPBBr_3$ nanobelts within the above constrains without any detectable material damage. In cases where we did not adhere to such constrains for example when selected area diffraction (SAD) pattern were obtained, clear beam damage is detected, see Fig. S2. The beam damage is seen in the TEM micrograph and in the SAD pattren in the form of extra diffraction spots and visible degradation of the perovskite. We find that the additional diffraction reflection that does not fit the $CsPbBr_3$ orthorhombic material, do fit metallic Pb (marked in red in Fig. S2a), in accordance with other studies that reported the degradation on the $CsPbBr_3$ in to Pb, CsBr and $PbBr_2$.

TEM image simulations

In the simulation process two software were used:

<u>TEM quantitative image simulation:</u> the image simulation was preformed using the QSTEM simulation software.[5] The simulation conditions were for TEM mode and acceleration voltage of 200kV. The data can be looked up at: https://www.physics.hu-berlin.de/en/sem/software/software_qstem

<u>Atomistic crystal model:</u> the perovskite atomistic crystal modal was built using the Samson software. The bent nanobelt was constructed by duplicating the unit cell and bending the nanobelt at the middle. The data can be looked up at: https://school.samson-connect.net/

Simulations of the buckled perovskite TEM images were done according to:

QSTEM- an open source quantitative TEM/STEM Simulations software which is based on the multislice algorithm. This software was built by Christoph Koch and is described in his PhD thesis done at Arizona State University. We used QSTEM to do 2 simulations:

- The first was simulation of planar perovskite crystal with variations in the angle compared to the substrate. In this simulation a cif. file of the $CsPbBr_3$ perovskite unit cell was uploaded to the software.
- The second image simulation was done using the same steps, but instead of uploading the cif. file of the unit cell and building it in the QSTEM software, we built a bent nanobelt in the Samson software and uploaded this file to the QSTEM software.

Samson- Software for computational nanoscience, it can rapidly build models of complex nano systems. This software was used to build an atomistic model of a bent nanobelt for the image simulations. The perovskite nanobelt was built using an additional element for crystals which is available in the Samson website. The crystal was built using a cif. file of a unit cell and manipulated in Samson.

Nanobelts buckling model and adhesion forces

The calculations of the adhesion forces were done based on standard elasticity theory. To model the buckled nanobelt we used a buckled plate model.

Plate buckling theory

Plate buckling theory expresses the stability of a plate under compressive load, usually it is presented by a critical force or stress, from which the plate will buckle. The expression of this critical force will depend on specific boundary conditions. A general expression of the critical force is:

$$(1)\ F_{cr} = \frac{\pi^2 Ewt}{({}^{L_e}/_{k})^2},$$

$$(2)\ k = \frac{t^2}{12},$$

Where E is Young's modulus, L_e is equivalent length, w is the width and t is thickness. In the $CsPbBr_3$ nanobelts the length of the contrast segment is in the range of 30-250 nm while the width of the nanobelt itself is 20-100 nm. This ratio between the length and the width of the buckled area corresponds to a plate. Furthermore, the rest of the unbuckled nanobelt is adhered to the substrate, making the ends of the buckled plate pinned, and though we can look at the contrast segment as a simply supported plate.

The equivalent length of a simply supported plate is:

$$(3)\ L_e = l(1 - v^2)^{1/2},$$

l is the length of the plate and υ is Poisson's ratio.

By substituting Eq. 2 and 3 in Eq. 1 we get:

$$(4)\ F_{cr} = \frac{\pi^2 Et^3 w}{12l^2(1 - v^2)},$$

The forces causing the buckling are capillary forces. The buckling is seen as contrast bands in the TEM micrographs after the sample has dried, indicating that there are adhesion forces between the nanobelt and the TEM grid holding the buckle in place. These adhesion forces must be larger than the critical buckling force in order to maintain the buckle. Therefore, the plate buckling critical force equation can describe the minimal adhesion force of the nanobelt:

$$(5)\ F_{adhesion} \cong \frac{\pi^2 Et^3 w}{12l^2(1 - v^2)},$$

In the calculation of the adhesion forces the Young's modulus was taken as 28 GPa based on the calculation done by Balestra et al.[6] This value is bigger than the Young's modulus reported for the bulk (15.8 GPa)[7], however at the nanoscale it was shown in other materials[8–15] that the Young's modulus can increase as the dimensions of the material decrease.

The thickness used in the calculation is t=9 nm. This parameter was chosen based on:

- The thickness of the nanobelts was measured as 2.5-10 nm by an AFM (Fig. S4).
- The PL measurements of the nanobelts exhibits an emission peak at 522 nm which is in agreement with the weak quantum confined $CsPbBr_3$ emission. This means that the smallest dimension of the nanobelt is bigger than the Bohr radios, which is bigger than 7 nm[16].
- In addition to the buckling seen as a contrast in the TEM micrographs (buckling out of the plane), we see nanobelts that buckle in plane (Fig. S9). In this situation the nanobelt can be presented as a buckled beam. The buckle sideways (in plane) instead of upwards (out of plane) indicates that the moment of inertia is smaller in that direction, because the buckling will be in the direction of the smallest moment of inertia. So, when the width of the nanobelt is bigger than the thickness an out of surface buckling will be favored, and when the width is smaller than the thickness the nanobelt will prefer to buckle in the plane of the surface. From statical analysis of the buckled nanobelts we concluded that the critical value is 9 nm.

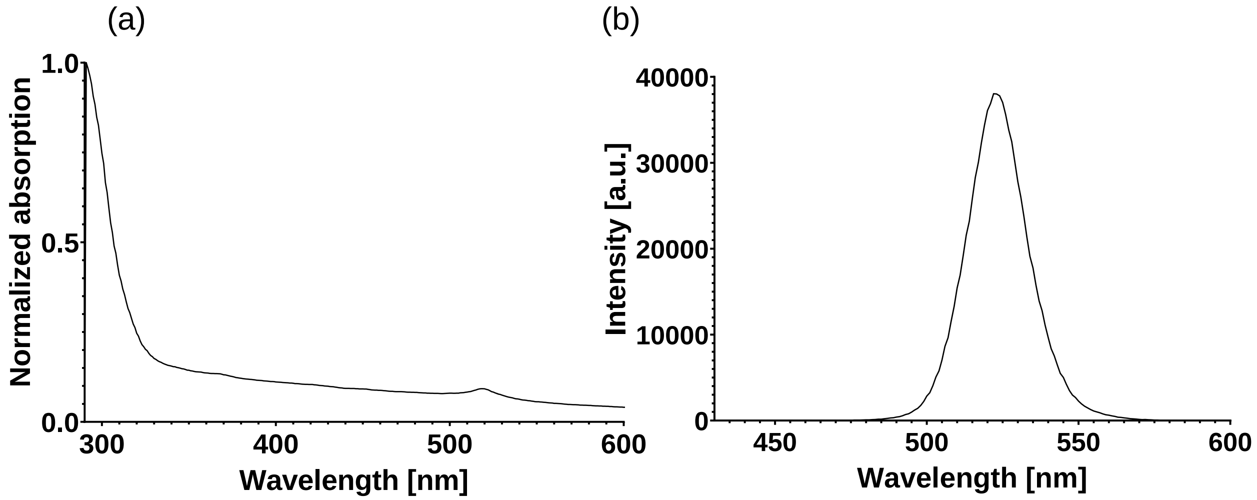

Figure S1. (a) Absorption and (b) PL spectra of the colloidal $CsPbBr_3$ nanobelts measured at room temperature matching the not quantum confined $CsPbBr_3$

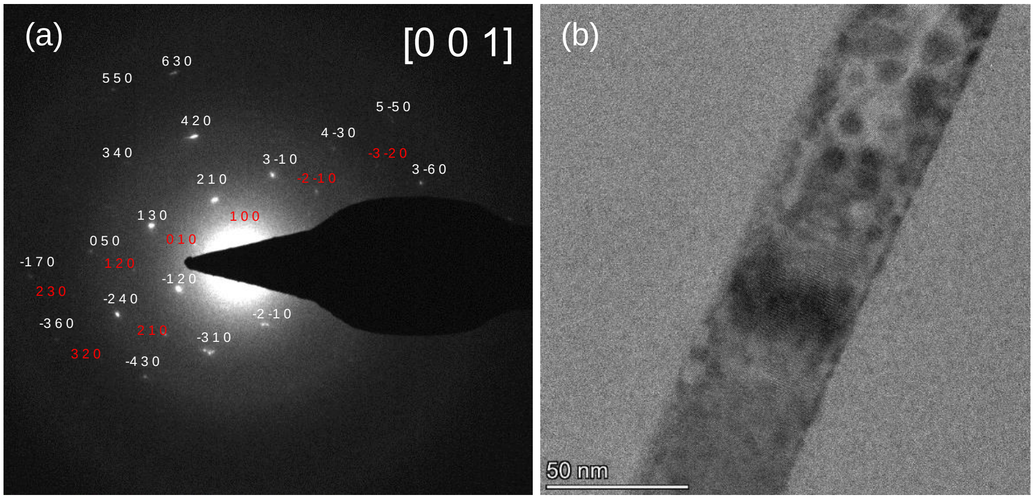

Figure S2. (a) Selected area diffraction pattern of the $CsPbBr_3$ nanobelts where long exposure and high magnification degrades the $CsPbBr_3$ (in white), and other diffraction reflections related to degradation products such as metallic Pb (in red) appear during the exposure. (b) Post diffraction HRTEM micrograph of the perovskite nanobelt depicting visible beam damges (higer contarst spots are metallic lead).

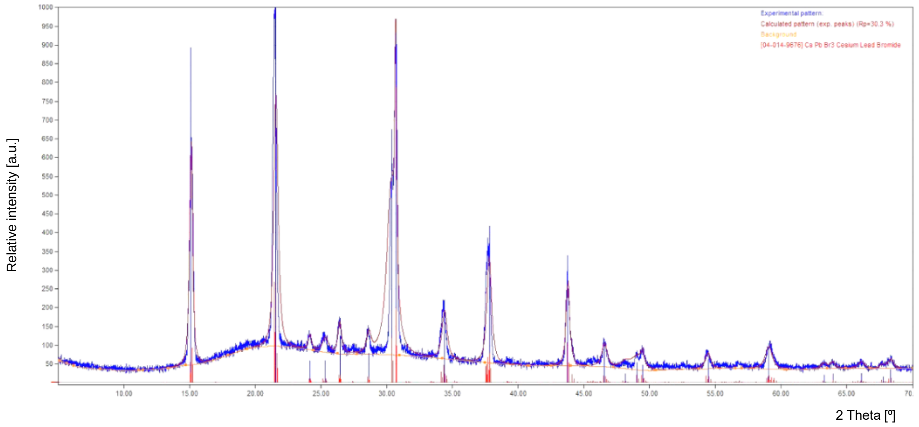

Figure S3. XRD analysis of the nanobelts (blue) showing an orthorhombic structure . Our data matches the JCPDS card 04-014-9676 of an orthorhombic $CsPbBr_3$ (red).

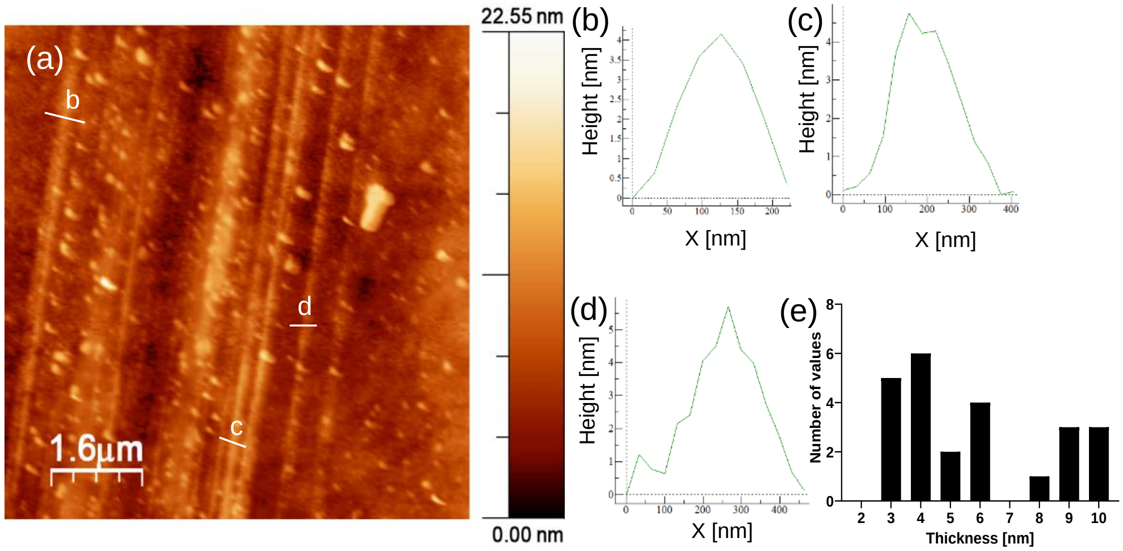

Figure S4. (a) AFM topography contrast of $CsPbBr_3$ nanobelts and thickness measured at 3 different locations (b),(c),(d). (e) A histogram of the nanobelts thickness measurments in the AFM.

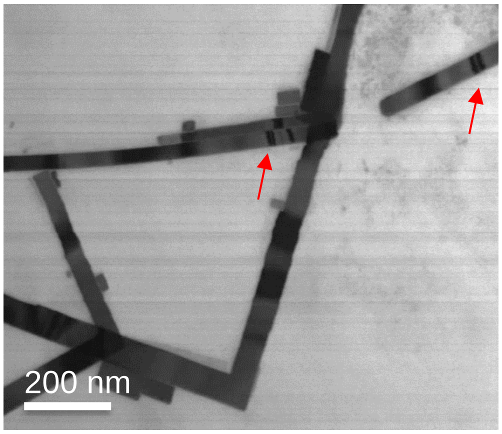

Figure S5. SEM micrograph of $CsPbBr_3$ nanobelts with bend contrasts indicated by the red arrows, similar to the TEM micrographs.

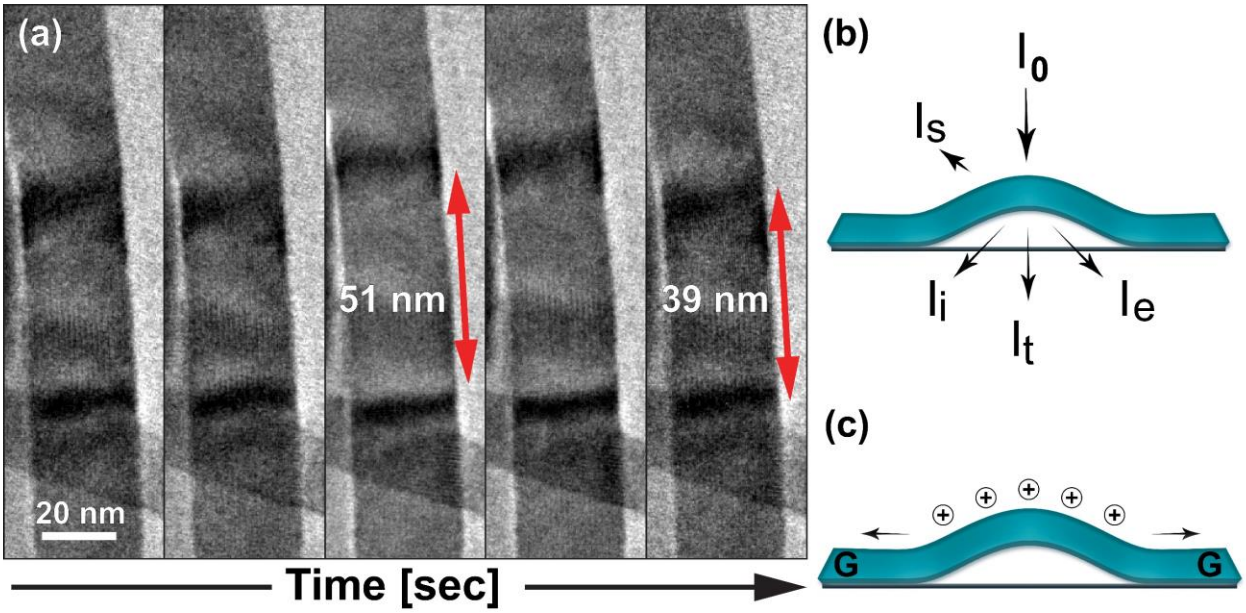

Figure S6. (a) Dynamic jitter of the contrast strip during TEM characterization. Appearance of the contrast changes while the sample is exposed to the TEM beam (~60 seconds) due to a dynamic charging effect. Distance between dark strips changes from maximum 51 nm to minimum 39 nm. (c)-(d) Illustration of potential charging of the buckled areas, charges accumulate on the buckled nanobelts due to lower electrical coupling to the conductive substrate.

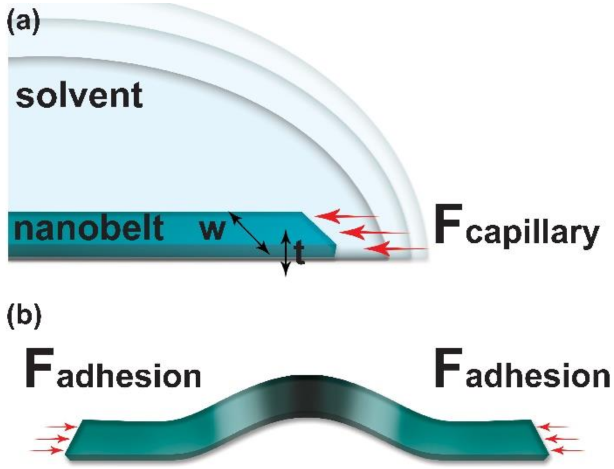

Figure S7. (a) A sketch of the capillary buckling forces acting on the nanobelt as part of the drying process. (b) A sketch of the buckled nanobelt with adhesion forces maintaining the buckle.

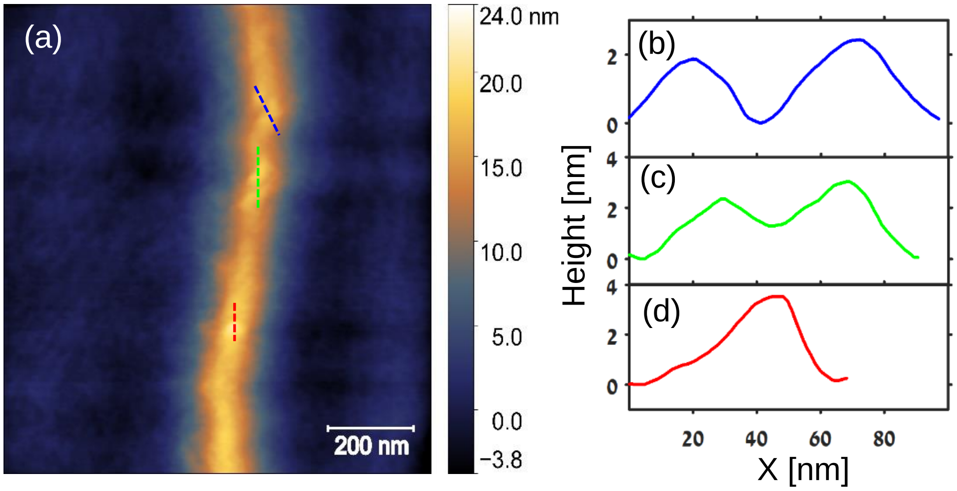

Figure S8. AFM analyses of a buckled nanobelt. (a) AFM micrograph of the nanobelt. (b) Topography measurement of the blue line. (c) Topography measurement of the green line. (d) Topography measurement of red line.

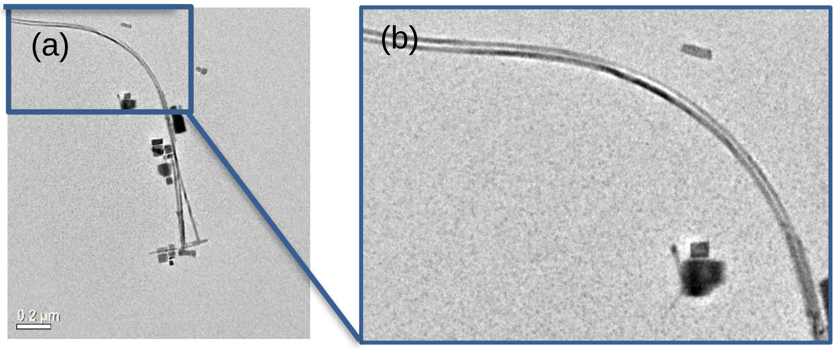

Figure S9. TEM micrograph of in plane nanobelt buckling. (a) A 10 nm wide nanobelts with an in plane buckling. (b) Zoom in of the selected area in a.

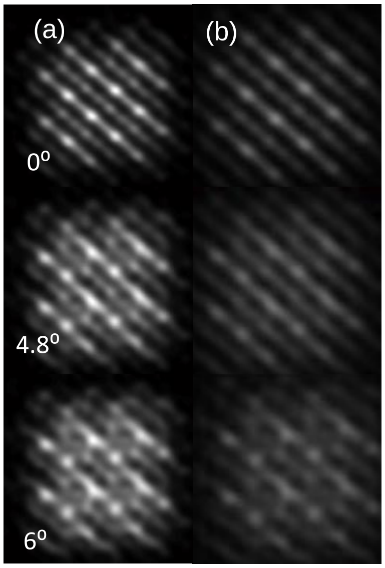

Figure S10. (a) Quantitative TEM simulated images, based on a multislice algorithm of tilted $CsPbBr_3$ crystals (α=0,4.8,6°). (b) FFT filtered images of the Quantitative TEM simulated images in (a).

REFERENCES

(1) Liu, Y.; Guo, M.; Dong, S.; Jiao, X.; Wang, T.; Chen, D. Room Temperature Colloidal Synthesis of CsPbBr 3 Nanowires with Tunable Length, Width and Composition. *J. Mater. Chem. C* **2018**, *6* (29), 7797–7802.

(2) Dang, Z.; Shamsi, J.; Palazon, F.; Imran, M.; Akkerman, Q. A.; Park, S.; Bertoni, G.; Prato, M.; Brescia, R.; Manna, L. In Situ Transmission Electron Microscopy Study of Electron Beam-Induced Transformations in Colloidal Cesium Lead Halide Perovskite Nanocrystals. *ACS Nano* **2017**, *11* (2), 2124–2132.

(3) Dang, Z.; Shamsi, J.; Akkerman, Q. A.; Imran, M.; Bertoni, G.; Brescia, R.; Manna, L. Low-Temperature Electron Beam-Induced Transformations of Cesium Lead Halide Perovskite Nanocrystals. *ACS Omega* **2017**, *2* (9), 5660–5665.

(4) Chen, X.; Wang, Z. Investigating Chemical and Structural Instabilities of Lead Halide Perovskite Induced by Electron Beam Irradiation. *Micron* **2019**, *116* (April 2018), 73–79.

(5) Koch, C. Determination of Core Structure Periodicity and Point Defect Density Along Dislocations, PhD thesis, Arizona state university, 2002.

(6) Rees, D. W. A. Plate Buckling Under Uniaxial Compression. In *Mechanics of Optimal Structural Design: Minimum Weight Structures*; John Wiley & Sons, 2009; pp 525–535.

(7) Rakita, Y.; Cohen, S. R.; Kedem, N. K.; Hodes, G.; Cahen, D. Mechanical Properties of APbX3 (A = Cs or CH3NH3; X= I or Br) Perovskite Single Crystals. *MRS Commun.* **2015**, *5* (4), 623–629.

(8) Wang, G.; Li, X. Predicting Young's Modulus of Nanowires from First-Principles Calculations on Their Surface and Bulk Materials. *J. Appl. Phys.* **2008**, *104* (11), 113517.

(9) Chen, C. Q.; Shi, Y.; Zhang, Y. S.; Zhu, J.; Yan, Y. J. Size Dependence of Young's Modulus in ZnO Nanowires. *Phys. Rev. Lett.* **2006**, *96* (7), 075505.

(10) Wang, C.; Wu, S.; Yang, X.; Yan, Z.; Xie, G.; Zhang, S.; Wang, J.; Cao, H. Thickness-Dependent Young's Modulus of Polycrystalline α-PbO Nanosheets. *Nanotechnology* **2020**,

31 (39).

(11) Agrawal, R.; Peng, B.; Gdoutos, E. E.; Espinosa, H. D. Elasticity Size Effects in ZnO Nanowires−A Combined Experimental-Computational Approach. *Nano Lett.* **2008**, *8* (11), 3668–3674.

(12) Zhu, Y.; Qin, Q.; Xu, F.; Fan, F.; Ding, Y.; Zhang, T.; Wiley, B. J.; Wang, Z. L. Size Effects on Elasticity, Yielding, and Fracture of Silver Nanowires: In Situ Experiments. *Phys. Rev. B* **2012**, *85* (4), 045443.

(13) Tan, E. P. S.; Zhu, Y.; Yu, T.; Dai, L.; Sow, C. H.; Tan, V. B. C.; Lim, C. T. Crystallinity and Surface Effects on Young's Modulus of CuO Nanowires. *Appl. Phys. Lett.* **2007**, *90* (16), 1–4.

(14) Wang, Y.-B.; Wang, L.-F.; Joyce, H. J.; Gao, Q.; Liao, X.-Z.; Mai, Y.-W.; Tan, H. H.; Zou, J.; Ringer, S. P.; Gao, H.-J.; Jagadish, C. Super Deformability and Young's Modulus of GaAs Nanowires. *Adv. Mater.* **2011**, *23* (11), 1356–1360.

(15) McDowell, M. T.; Leach, A. M.; Gall, K. Bending and Tensile Deformation of Metallic Nanowires. *Model. Simul. Mater. Sci. Eng.* **2008**, *16* , 045003 (13pp).

(16) Protesescu, L.; Yakunin, S.; Bodnarchuk, M. I.; Krieg, F.; Caputo, R.; Hendon, C. H.; Yang, R. X.; Walsh, A.; Kovalenko, M. V. Nanocrystals of Cesium Lead Halide Perovskites (CsPbX 3 , X = Cl, Br, and I): Novel Optoelectronic Materials Showing Bright Emission with Wide Color Gamut. *Nano Lett.* **2015**, *15* (6), 3692–3696.
